# Using Bayesian statistics for modeling PTSD through Latent Growth Mixture Modeling: implementation and discussion

**DOI:** 10.3402/ejpt.v6.27516

**Published:** 2015-03-02

**Authors:** Sarah Depaoli, Rens van de Schoot, Nancy van Loey, Marit Sijbrandij

**Affiliations:** 1 Psychological Sciences, University of California, Merced, CA, USA; 2 Department of Methods and Statistics, Utrecht University, Utrecht, The Netherlands; 3 Optentia Research Program, Faculty of Humanities, North-West University, Mahikeng, South Africa; 4 Department of Clinical and Health Psychology, Utrecht University, Utrecht, The Netherlands; 5 Department of Behavioral Research, Association of Dutch Burns Centres, AJ Beverwijk, The Netherlands; 6 Clinical Psychology, VU University, Amsterdam, The Netherlands; 7 EMGO Institute for Health and Care Research, Amsterdam, The Netherlands

**Keywords:** Latent Growth Mixture Modeling, Bayesian estimation, sensitivity analysis, PTSD, delayed onset

## Abstract

**Background:**

After traumatic events, such as disaster, war trauma, and injuries including burns (which is the focus here), the risk to develop posttraumatic stress disorder (PTSD) is approximately 10% (Breslau & Davis, 1992). Latent Growth Mixture Modeling can be used to classify individuals into distinct groups exhibiting different patterns of PTSD (Galatzer-Levy, 2015). Currently, empirical evidence points to four distinct trajectories of PTSD patterns in those who have experienced burn trauma. These trajectories are labeled as: resilient, recovery, chronic, and delayed onset trajectories (e.g., Bonanno, 2004; Bonanno, Brewin, Kaniasty, & Greca, 2010; Maercker, Gäbler, O'Neil, Schützwohl, & Müller, 2013; Pietrzak et al., 2013). The delayed onset trajectory affects only a small group of individuals, that is, about 4–5% (O'Donnell, Elliott, Lau, & Creamer, 2007). In addition to its low frequency, the later onset of this trajectory may contribute to the fact that these individuals can be easily overlooked by professionals. In this special symposium on Estimating PTSD trajectories (Van de Schoot, 2015a), we illustrate how to properly identify this small group of individuals through the Bayesian estimation framework using previous knowledge through priors (see, e.g., Depaoli & Boyajian, 2014; Van de Schoot, Broere, Perryck, Zondervan-Zwijnenburg, & Van Loey, 2015).

**Method:**

We used latent growth mixture modeling (LGMM) (Van de Schoot, 2015b) to estimate PTSD trajectories across 4 years that followed a traumatic burn. We demonstrate and compare results from traditional (maximum likelihood) and Bayesian estimation using priors (see, Depaoli, 2012, 2013). Further, we discuss where priors come from and how to define them in the estimation process.

**Results:**

We demonstrate that only the Bayesian approach results in the desired theory-driven solution of PTSD trajectories. Since the priors are chosen subjectively, we also present a sensitivity analysis of the Bayesian results to illustrate how to check the impact of the prior knowledge integrated into the model.

**Conclusions:**

We conclude with recommendations and guidelines for researchers looking to implement theory-driven LGMM, and we tailor this discussion to the context of PTSD research.
